# Editorial: Advances in the Biology and Medicine of Pain

**DOI:** 10.3389/fnins.2022.970635

**Published:** 2022-07-26

**Authors:** Alexandra Latini, Michael Costigan

**Affiliations:** ^1^LABOX, Department of Biochemistry, Universidade Federal de Santa Catarina, Florianópolis, Brazil; ^2^Harvard Medical School, Boston Children's Hospital, Boston, MA, United States

**Keywords:** biomarker, physiopathology, clinical trial, drug discovery, neuropharmacology

Chronic pain is a disease of nociceptive circuits at any level of the nervous system, characterized by abnormal sensitivity to thermal and mechanical stimuli. Hypersensitivity can be expressed as hyperalgesia, an excessive reaction to normally painful input; allodynia, a painful response to normally innocuous stimuli; or spontaneous pain with no identifiable cause. Furthermore, non-neuronal cells may also contribute to the physiopathology of chronic physical and psychosocial changes such as disability, anxiety, depression, and disturbed sleep. These secondary symptoms, in addition to the persistent pain itself, often have a devastating impact on the patient's quality of life.

Chronic pain is estimated to be one of the most prevalent health problems in the world, with almost all of us dealing with it at some point in our lives. Although scientists have made great advances in the understanding of the molecular mechanisms through which persistent pain develops, this knowledge has not been translated into safe and effective therapies. Indeed, current gold-standard analgesics for chronic pain management have limited efficacy in the majority of patients while producing many side effects, and in the case of opioids, they have a high liability for abuse with a high risk of death. Therefore, the management of chronic pain is a major unmet clinical need and the lack of effective treatment places an immense burden on patients, families, health-care systems and society in general. As a result, several drug discovery groups are committed to finding new pharmacological and non-pharmacological avenues to manage chronic pain.

The Research Topic *Advances in the Biology and Medicine of Pain* includes 34 articles that address different aspects of chronic pain's physiopathology, treatment, identification of predictors of pain and a clinical trial. Most of the reports are pre-clinical studies, but they also include some clinical research. In addition, readers will also find several reviews addressing the participation of the immune response, stress, and different metabolic pathways in favoring the developing of chronic pain. All these articles create a cohesive understanding of recent advances and future directions in the pathophysiology and treatment of persistent pain.

## Overview of the Articles Included in Advances in the Biology and Medicine of Pain

The reviews presented in this Research Topic highlight the need to develop new therapies for pain management, stressing that a deeper understanding of mechanisms of painful sensitization is essential, considering that so far, there is not a safe and effective way to treat chronic pain.

Manion et al. revised the recent progress in the understanding of mechanisms underlying pain, and how these mechanisms are being targeted to produce modern, specific therapies for pain. In this scenario, various authors addressed how metabolic pathways can contribute to the transition from acute to chronic pain and how their metabolic intermediates might be potential biomarkers for chronic pain and its treatment. Staats Pires et al. collected evidence showing that the kynurenine and the tetrahydrobiopterin pathways can be modulated by the use of metabolic inhibitors representing novel ways to treat chronic pain. Osthues and Sisignano also discussed that oxidized lipid intermediates, as well as some lysophospholipids, sphingolipids, and specialized pro-resolving mediators may play distinct roles in pain modulation, proposing these pathways as potential targets for the development of new analgesics. Likewise, Quintão et al.'s study focused on the mechanisms involved in chemotherapy-induced neuropathy (CINP) and how activators of peroxisome proliferator-activated receptors (PPAR), in particular PPAR-g signaling, can enhance the cell's antioxidant status and mitochondrial activity, two pathways compromised in CINP. Moreover, Bravo et al. provided preclinical and clinical evidence of the potential role of monoamines in the modulation of chronic pain, reviewing how this system is implicated in the analgesic mechanism of action of antidepressants, gabapentinoids, atypical opioids, NSAIDs and histaminergic drugs. Two other articles describe interventions aimed at preventing the transition from acute to chronic pain by modulating the activation of cytotoxic immune effector cells in injured peripheral nerves (Davies et al.) and by compromising the neuro-immune crosstalk, in particular in obesity where the state of chronic low-grade inflammation might heighten sensory hypersensitivity (Eichwald and Talbot). In addition, Lunde and Sieberg proposed a pain-stress model highlighting that a greater insight into the neurobiology of stress might contribute to individualized treatment for pain rehabilitation and drug development, since stress and chronic pain are chronic processes.

The systematic reviews presented in this Research Topic evaluated the analgesic effects of several drugs and procedures on different painful conditions. Xu and Sun found that calcitonin gene-related peptide receptor (CGRPR) antagonists are promising compounds for the acute treatment of migraine, especially in patients who are unable to take triptans. In addition, they found that olcegepant was the most effective and that ubrogepant had the lowest toxicity among six different CGRPR antagonists. Similarly, Zhu et al. evaluated the efficacy of intravenous lidocaine compared with the placebo for the management of neuropathic pain and the safety of its administration. The authors found that while the treatment is effective in pain control in the immediate post-infusion period, it does not have a long-lasting persistent effect. Indeed, the infusion of this drug was associated with increased risk of side effects. Regarding non-pharmacological therapies, it was found that the top-down neuromodulator effects of transcranial direct current stimulation is a promising approach to improve management in refractory chronic non-cancer-related pain and to enhance dysfunctional neuronal circuitries involved in the dysfunctional neuroplasticity induced by opioids (Zortea et al.). Furthermore, Lima et al. found reduced pain scores induced by manual therapy in different pre-clinical reports that mimicked neuromusculoskeletal diseases. The authors stressed that understanding the mechanisms responsible for the analgesic effect of this procedure will allow for more insight into the physiopathology of chronic pain and for designing complex and longitudinal studies with appropriate controls by using, e.g., larger animal models (sheep, pig, etc.), which biomechanically are more similar to what is observed in the clinical setting. Similarly, the study by Gunduz et al. aimed to bring evidence that the cortical reorganization induced after an amputation might be a potential clinical target for prevention and treatment response of phantom limb pain.

One article of the present Research Topic performed a prospective, randomized, double-blind, sham-controlled, proof-of-concept clinical trial in order to investigate the effect of transcranial direct current stimulation vs. sham stimulation on postoperative morphine consumption and pain intensity after thoracotomy. The authors showed that the sessions significantly reduced cumulative postoperative morphine use and pain scores without obvious long-term benefits (Stamenkovic et al.). Another clinical study demonstrated that facet joint injections of anesthetic and corticosteroids are useful for the diagnosis and treatment of lower back pain. It also showed that pain-related cognitive and behavioral factors determined by pain catastrophizing and smoking are independently associated with pain recurrence. The authors pointed out the need for a multidisciplinary approach toward pre-surgical evaluation of patients with chronic pain (Campos et al.).

Among the original research, several articles focused on the attenuation of inflammation-induced pain by targeting specific proteins. Wang et al. showed that the intrathecal administration of maresin 1, a newly discovered pro-resolving lipid mediator, reduced radicular pain by inhibiting NLRP3 inflammasome-induced pyroptosis *via* NF-kB signaling in rodents. Ni et al. showed that the intrathecal administration of liquiritin alleviated mechanical allodynia in a rat model of bone cancer pain, by inhibiting the activation of the C-X-C motif chemokine ligand (CXCL) 1/CXCR2 signaling pathway and the production of IL-1β and IL-17. Pitake et al. found increased activity of voltage-gated calcium channels during inflammation, eliciting thermal hyperalgesia through both, changes in firing rates of sensory neurons as well as the promotion of new neurite outgrowth in mice. By using the same administration route, it was also shown that aminoglutethimide, an inhibitor of the neurosteroidogenic enzyme, cytochrome P450 side-chain cleavage enzyme, reduced its expression and increased D-serine immunoreactivity during the induction phase of an animal model of neuropathic pain (Choi et al.). Another pain-related pathway was investigated by Xu et al. who showed that 17β-estradiol reduced clinical pain scores by upregulating voltage-gated chloride channel-3 in the dorsal root ganglion (DRG) of ovariectomized rats. The participation of artemin, a neurotrophic factor, and its receptor, glial-derived neurotrophic factor family receptor alpha-3, in chronic pain was shown in tissues of naturally occurring osteoarthritis in dogs. In addition, increased levels of artemin from osteoarthritic humans were found, indicating a translational relevance of this pathway since it may ultimately lead to the development of novel therapeutics (Minnema et al.).

Chronic pain was also evaluated in animal models of chronic conditions that have inflammation as a key component of their physiopathology, including obesity and inflammatory bowel diseases. Cifani et al. showed that rats exposed to palatable foods developed increased pain thresholds; however, during abstinence pain hypersensitivity was elicited. This effect was ameliorated by the repeated treatment with a fatty acid amide hydrolase inhibitor, PF-3845, which also up-regulated the expression of mu-opioid receptors. Thus, the authors suggested that this cannabinoid modulation should be considered when pharmacologically managing chronic pain in subjects affected by obesity. In addition, Ko et al. showed that glutamatergic transmission in the ventrolateral periaqueductal gray mediates depression-like behaviors during remission of colitis-induced visceral pain.

The opioid-induced analgesia and hyperalgesia were also presented by several authors. The study by de Freitas et al. showed that central extracellular signal-regulated kinase was involved in the analgesic and hyperalgesic effects of a single dose of morphine, while c-Jun N-terminal kinase, p38, and cAMP response element-binding protein were involved in the morphine-induced delayed hyperalgesia. Also, a decreased analgesic responses to morphine induced by the inhibition of an ATP-sensitive potassium (KATP) channel in sensory neurons, the SUR1 KATP channel subtype, was demonstrated by Fisher et al. The authors proposed the use of inhibitors of this particular channel as a viable option to alleviate opioid tolerance and withdrawal. In this scenario, Huang et al. investigated the role of miRNAs in the development of morphine tolerance. The authors demonstrated that miR-873a-5p targets tumor necrosis factor a-induced protein 3 in the spinal cord, facilitating the development of morphine tolerance in mice; hence, its downregulation may become a potential strategy for ameliorating morphine tolerance. Balogh et al. also evaluated the reduction in the analgesic action of opioids in diabetic neuropathic pain and demonstrated that advanced diabetes impairs the antinociceptive effect of systemic morphine but not of the new opioid agonist 14-O-methymorphine-6-O-sulfate in rats.

Other original studies focused on nociceptive molecular mechanisms, demonstrating that topical capsaicin, used in clinics with the objective of temporarily desensitizing epidermal nociceptors, selectively impaired heat sensitivity without any concomitant changes in cold sensitivity in healthy volunteers (van Neerven and Mouraux). When looking for human pain biomarkers the original research of this topic showed that osteoarthritis' disease severity can be assessed by knee magnetic resonance imaging associated with type II collagen cleavage products levels (Sofat et al.). Basu et al. demonstrated that Euphorbia bicolor latex phytochemicals induced hyperalgesia followed by peripheral, non-opioid analgesia in rats, which occurred in part *via* the transient receptor potential V1 ion channel. This is the first report on the analgesic properties of phytochemicals of the genus Euphorbia bicolor. Also, Schwertner et al. explored the effects of S-ketamine on the affective aspect of interpretation of stimuli using event-related potentials. The authors found evidence of changes in the interpretation of pain-related words in both neurophysiological and behavioral outcomes. S-ketamine induced a state of emotional and discrimination blunting when compared to placebo.

Non-pharmacological strategies to manage chronic pain were also presented in this Research Topic. Spinal mobilization is one of the most recommended practices for treating lower back pain; however, the molecular mechanisms involved in the analgesic effects are virtually unknown. Reed et al. demonstrated that the nerve growth factor-induced mechanical hyperalgesia and distant allodynia may be counterbalanced by spinal mobilization by attenuating the expression of calcitonin gene-related peptide in lumbar DRG sensory neurons.

Finally, it was demonstrated that the pain numerical rating scale, which is widely used in pain research and clinical settings to quantify pain intensity did not provide a reliably interpretable assessment of physical pain intensity for an individual with chronic low back pain at a specific moment (Griffin et al.).

## Author Contributions

AL and MC conceived and designed the Research Topic. All authors approved the final version of the manuscript.

## Funding

This work was supported by Fundação de Amparo à Pesquisa e Inovação do Estado de Santa Catarina (FAPESC/PPSUS 2021TR000452) and Conselho Nacional de Desenvolvimento Científico e Tecnológico (CNPq/Universal 422488/2021-6). AL was a CNPq fellow.

## Conflict of Interest

The authors declare that the research was conducted in the absence of any commercial or financial relationships that could be construed as a potential conflict of interest.

## Publisher's Note

All claims expressed in this article are solely those of the authors and do not necessarily represent those of their affiliated organizations, or those of the publisher, the editors and the reviewers. Any product that may be evaluated in this article, or claim that may be made by its manufacturer, is not guaranteed or endorsed by the publisher.

